# Supplementary figures and images for: Improved effectiveness of vaccination campaigns against rabies by reducing spatial heterogeneity in coverage
Source: PLoS Biol. 2025 May 5;23(5):e3002872. doi: 10.1371/journal.pbio.3002872 (PMC12068718; doi:10.1371/journal.pbio.3002872)

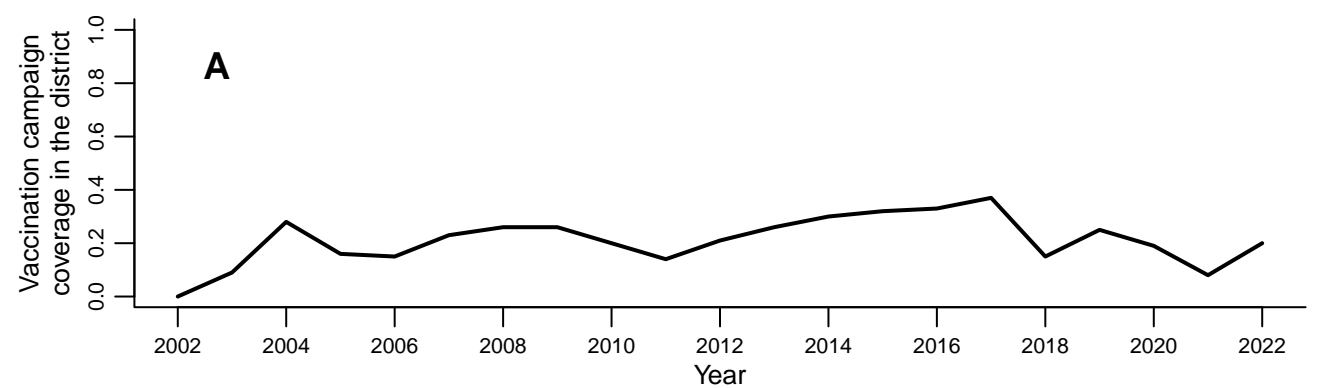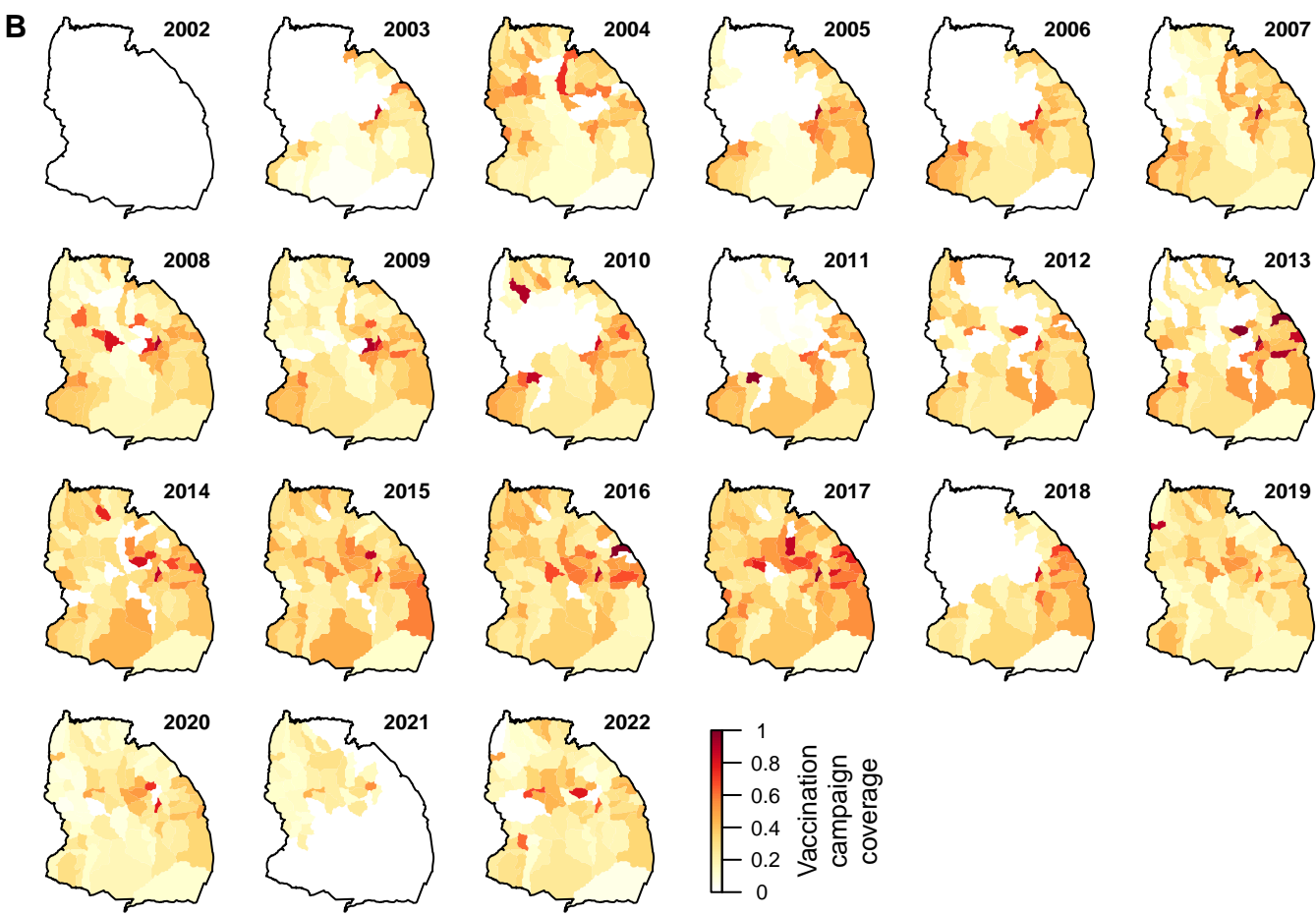

Supplement: S1 Fig — Map of Serengeti villages developed by [41] and available at https://doi.org/10.5281/zenodo.6308051. The data underlying this figure can be found at https://doi.org/10.5281/zenodo.15012106. (PDF) [file pbio.3002872.s002.pdf]

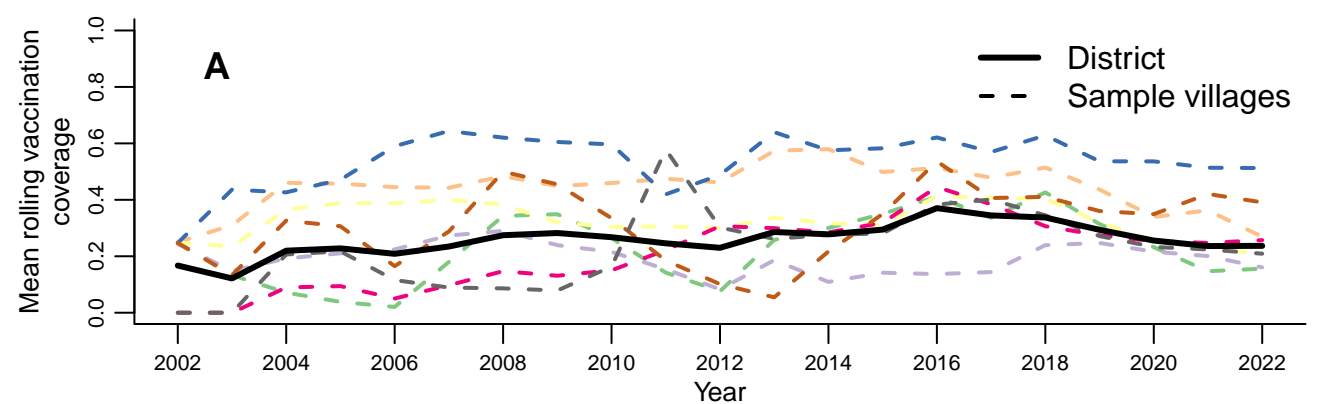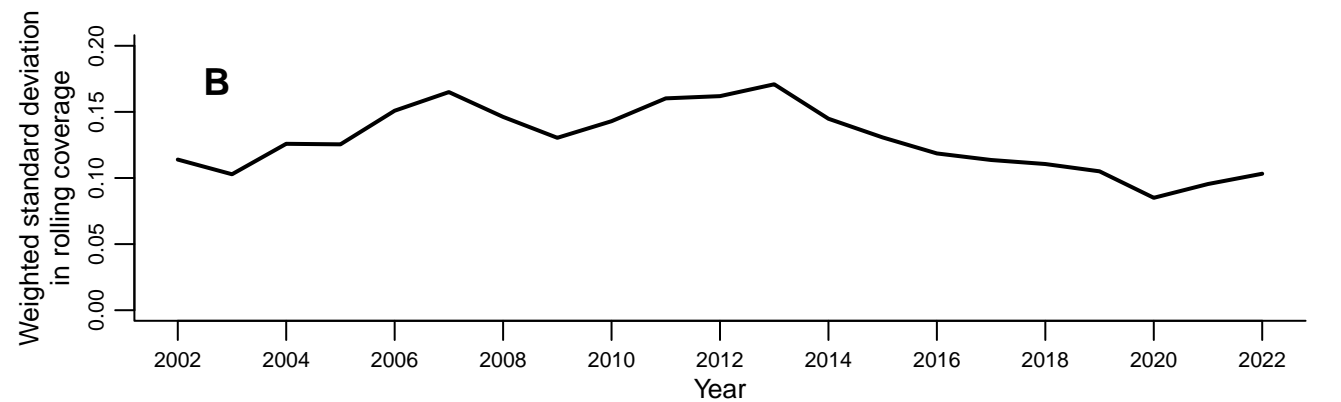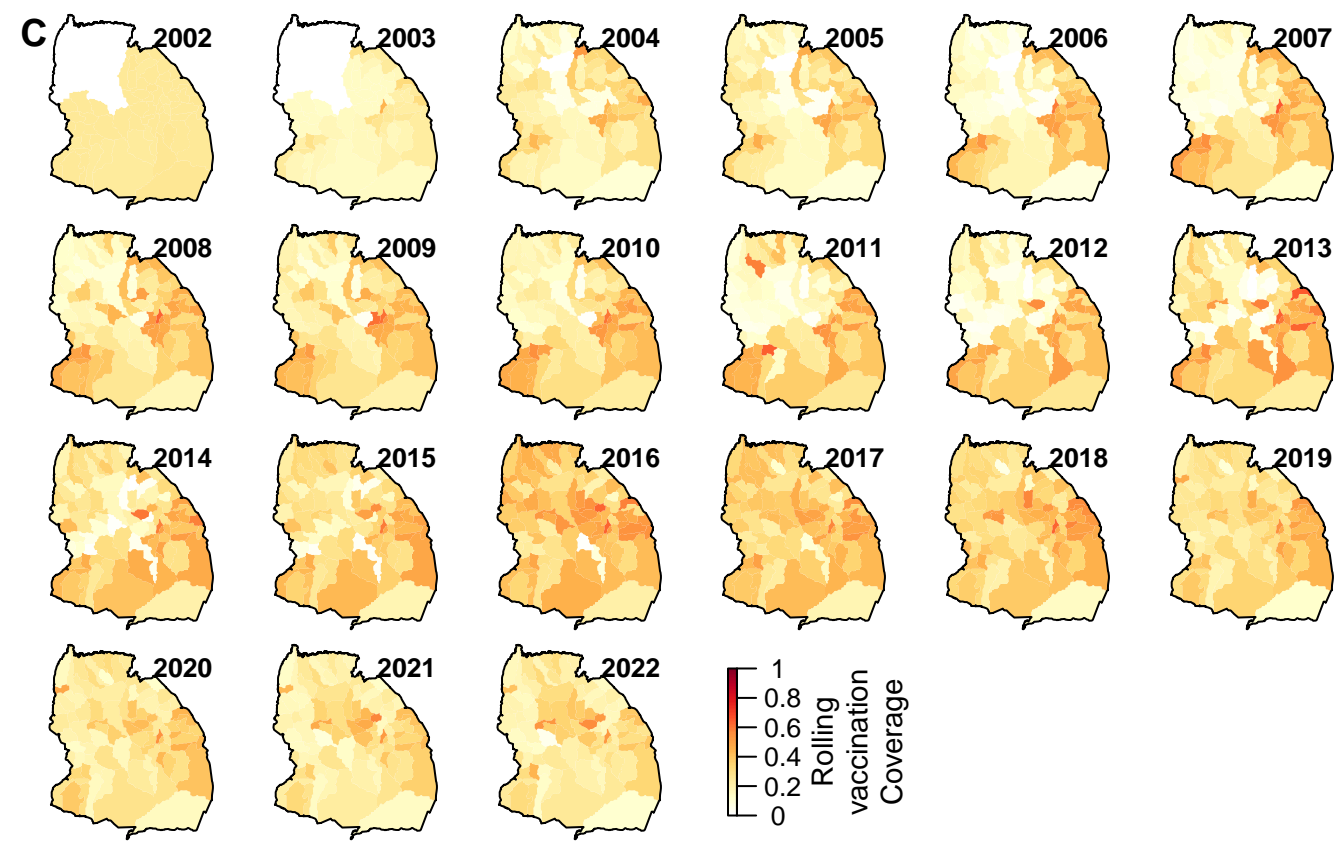

Supplement: S2 Fig — (A) The mean rolling vaccination coverage in Serengeti District over each year (12-month averages of the values in Fig 2C) is indicated by the solid black line. Mean rolling coverages each year for 8 randomly selected villages is indicated by dashed colored lines. (B) The weighted standard deviation in the rolling vaccination coverage over the villages in Serengeti District (yearly means of the values in Fig 2C). (C) Mean rolling vaccination coverage in each village over each year from 2002 to 2022 is indicated by the color scale. Map of Serengeti villages developed by [41] and available at https://doi.org/10.5281/zenodo.6308051. The data underlying this figure can be found at https://doi.org/10.5281/zenodo.15012106. (PDF) [file pbio.3002872.s003.pdf]

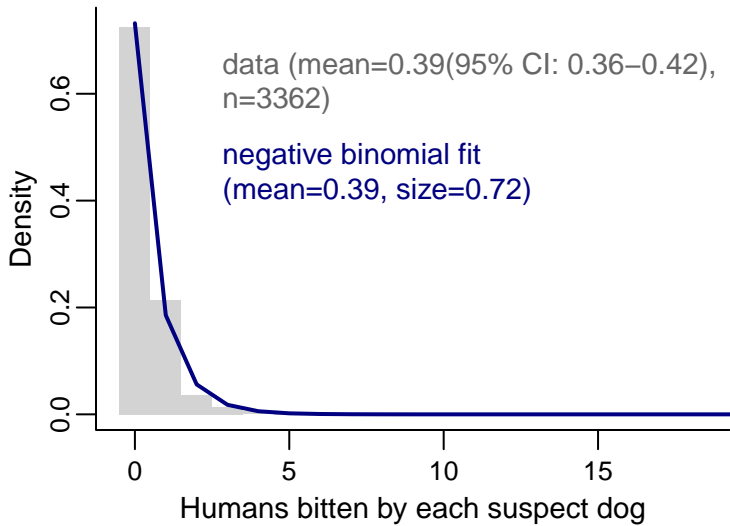

Supplement: S3 Fig — Histogram of human rabies exposures by each rabid dog from contact tracing data (grey bars), with fitted negative binomial distribution (blue line). The data underlying this figure can be found at https://doi.org/10.5281/zenodo.15012106. (PDF) [file pbio.3002872.s004.pdf]

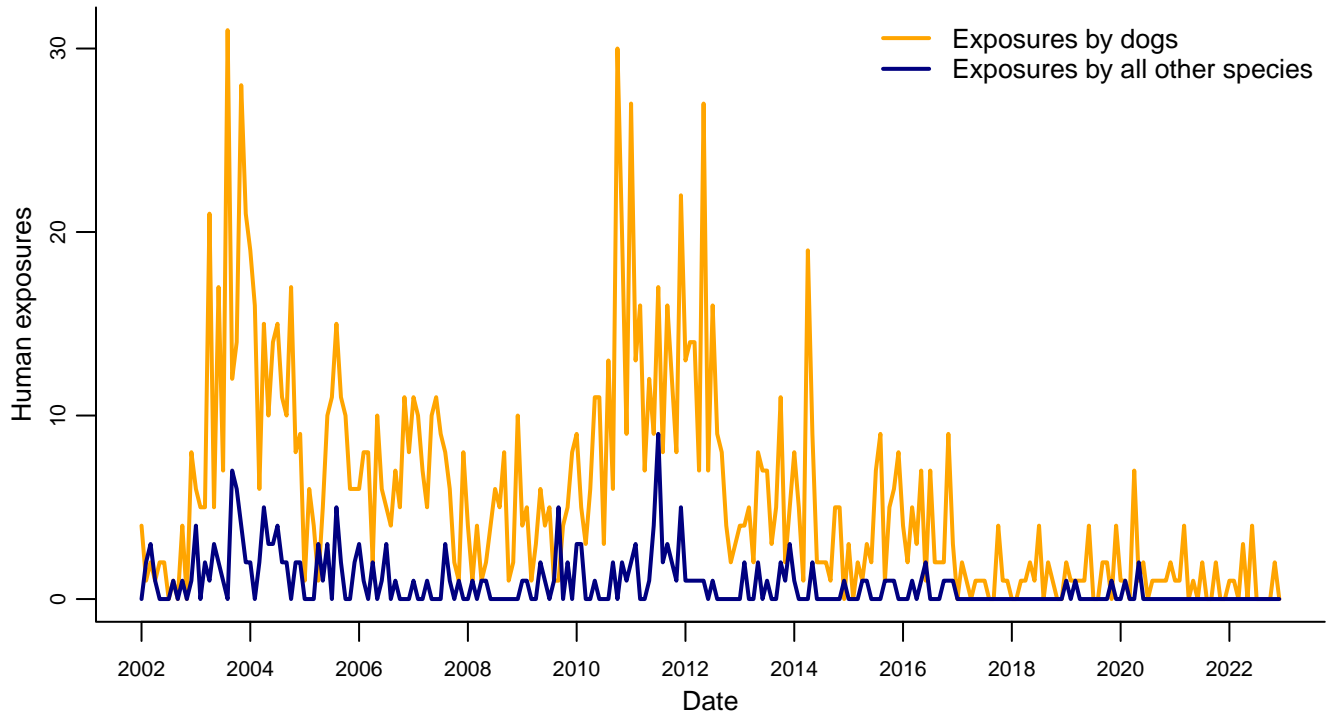

Supplement: S4 Fig — The data underlying this figure can be found at https://doi.org/10.5281/zenodo.15012106. (PDF) [file pbio.3002872.s005.pdf]

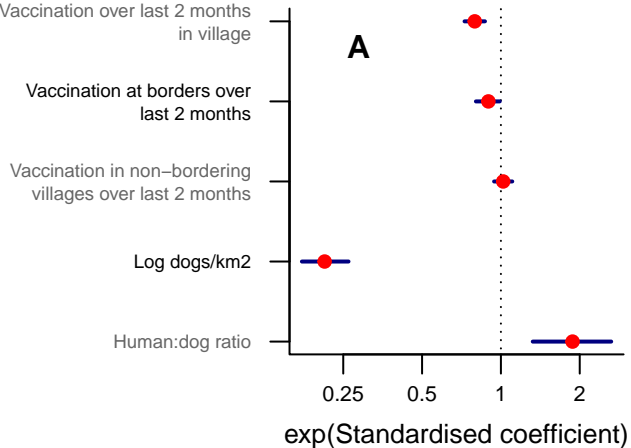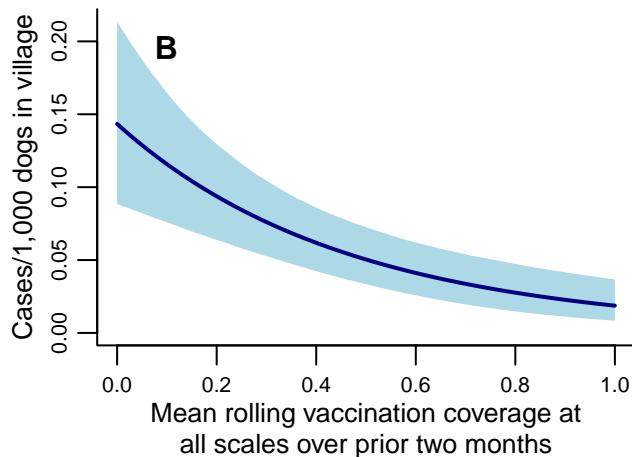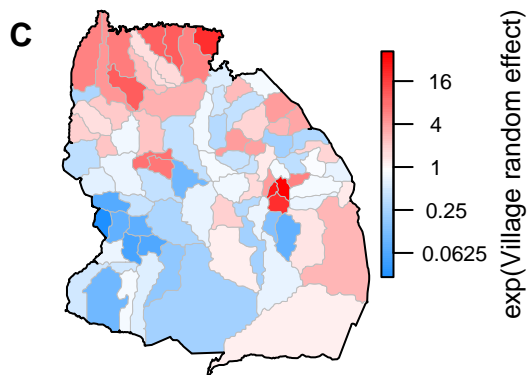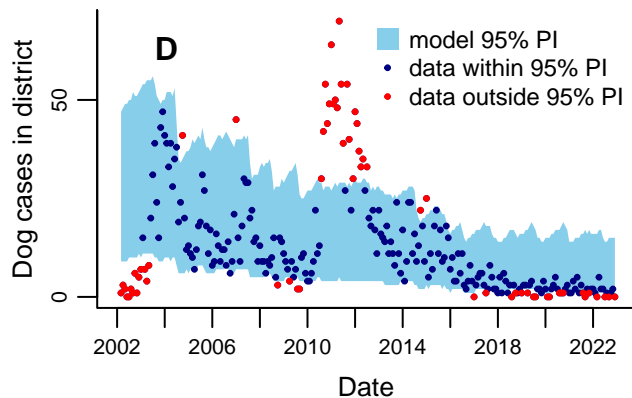

Supplement: S5 Fig — (A) Exponentiated standardized values of the coefficients estimated for each explanatory variable, with 95% CrIs. (B) Line shows the expected cases/1,000 dogs (number of dog cases normalized by dog population) in a village this month for different mean rolling vaccination coverages across the focal village and district in the prior 2 months. Shaded areas show 95% credible intervals (CrIs), and predictions were obtained using average values of unspecified explanatory variables. (C) Exponentiated random effect values for each village in the district. (D) Comparison of observed monthly dog cases (points) with the 95% prediction interval from the fitted model. Data points in red fall outside the 95% prediction interval (PI). Map of Serengeti villages developed by [41] and available at https://doi.org/10.5281/zenodo.6308051. The data underlying this figure can be found at https://doi.org/10.5281/zenodo.15012106. (PDF) [file pbio.3002872.s006.pdf]

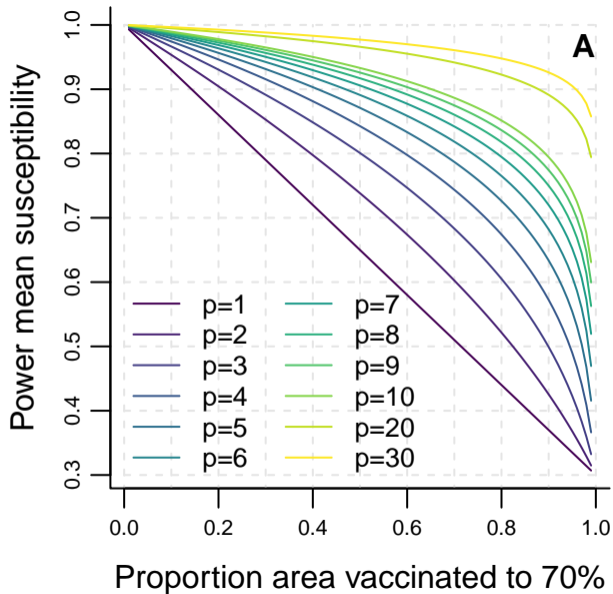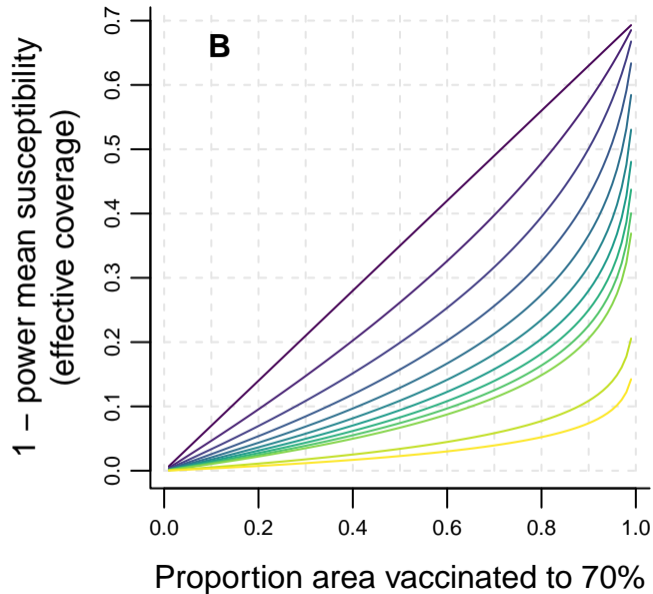

Supplement: S6 Fig — Here we assume a landscape where an increasing proportion of the area is vaccinated to 70% (30% susceptibility) while the remaining proportion remains at 0% coverage (100% susceptibility). We then calculate (A) the power mean susceptibility and (B) the effective coverage (1 – power mean susceptibility) at each proportion of area vaccinated at a range of values of the power p (Equation (14)). p = 1 is the arithmetic mean, and represents a scenario where, for a given proportion of dogs being vaccinated, the effective level of vaccination is the same regardless of how these vaccinated dogs are distributed over the area, i.e., heterogeneity in vaccination does not reduce (or increase) the impact of that vaccination on rabies cases. We used these curves showing the impact of different powers to select the prior distribution for p~N(μ = 1, σ = 2). If 99% of the area is covered, then the arithmetic mean coverage is 69.3%. If p = 2, then the effective coverage for the heterogeneous landscape is 68.5%; 0.8% lower than if vaccination had been homogeneous. If p = 5, however, effective coverage would be 58.4%, which is 10.9% below the arithmetic mean, despite only 1% of the area being uncovered, which seems an excessively large effect. The choice of σ = 2 was therefore made to exclude p ≥ 5 from the a priori confidence interval. The data underlying this figure can be found at https://doi.org/10.5281/zenodo.15012106. (PDF) [file pbio.3002872.s007.pdf]

Cases/1,000 dogs in village from model:

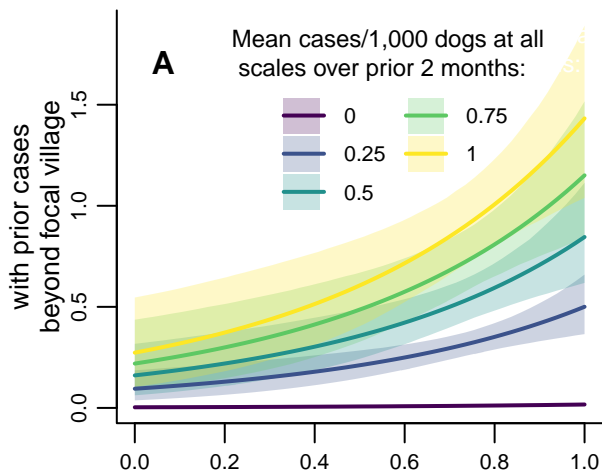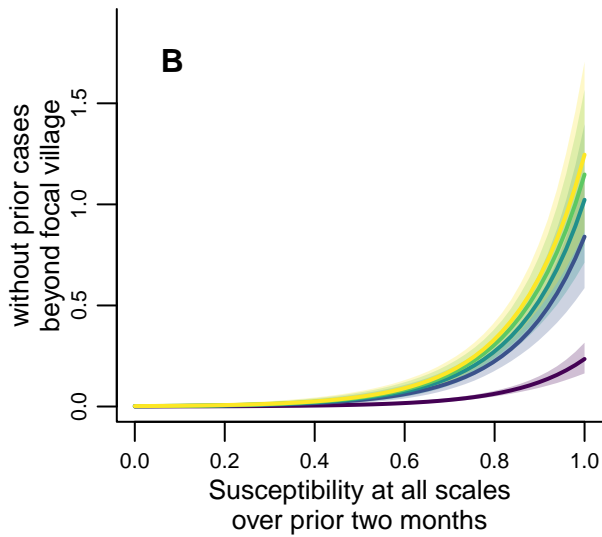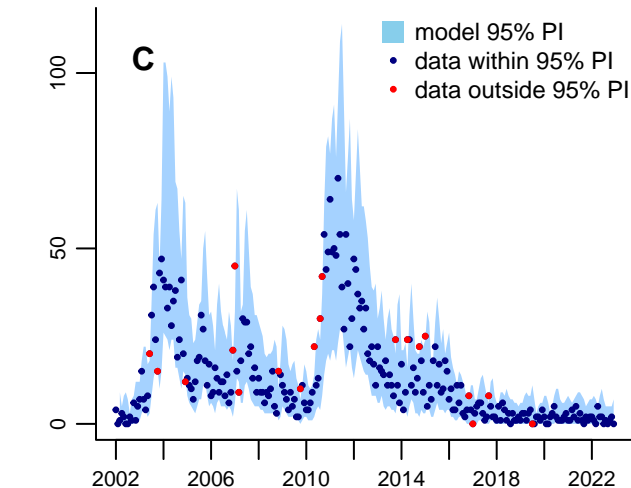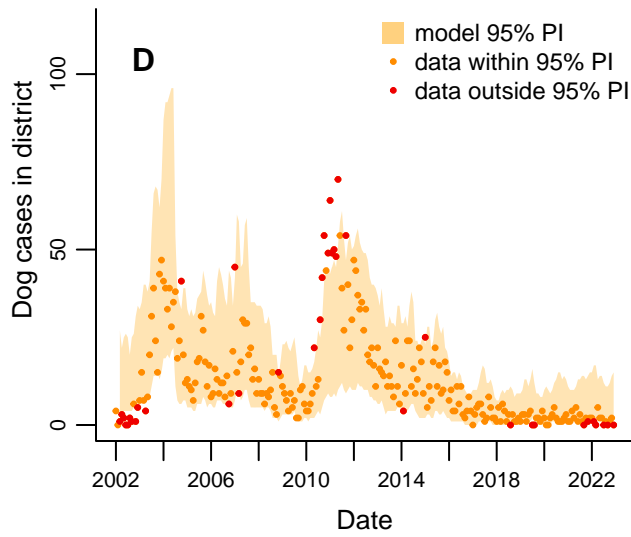

Supplement: S7 Fig — (A–B) Expected cases/1,000 dogs in a village from models with (A) and without (B) effects of prior incidence beyond the village. Predictions are shown for different mean susceptibilities (assuming homogeneous vaccination, i.e., power mean susceptibilities beyond the village equal susceptibility in the village) and mean cases/dog in the prior 2 months. Prior cases/dog values represent the observed district-level range and shaded areas show 95% CrIs, Predictions were obtained using average values of unspecified explanatory variables. (C–D) Comparison of observed monthly dog cases (points) with the 95% prediction interval from the fitted model with (C) or without (D) prior incidence beyond the village. Data points in red fall outside the 95% prediction interval (PI). The data underlying this figure can be found at https://doi.org/10.5281/zenodo.15012106. (PDF) [file pbio.3002872.s008.pdf]

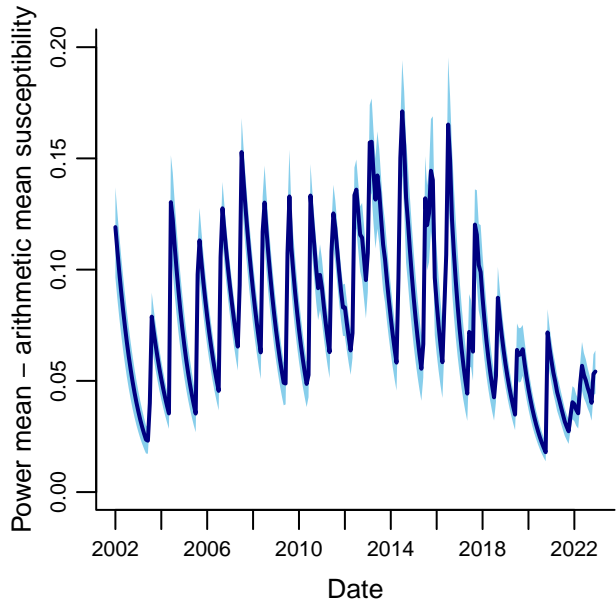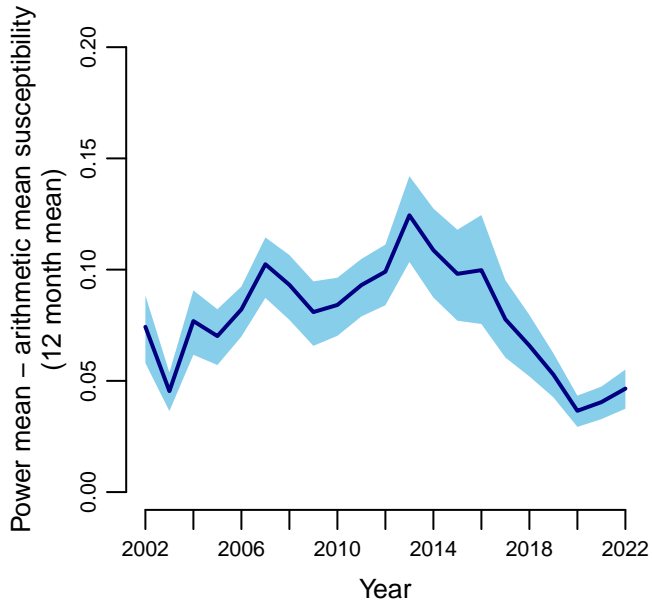

Supplement: S8 Fig — Difference between power mean susceptibility calculated over all villages in the district using fitted values of p from the model without prior incidence beyond the focal village (Fig 4C) minus the arithmetic mean for each month (A) or averaged over each year (B). The data underlying this figure can be found at https://doi.org/10.5281/zenodo.15012106. (PDF) [file pbio.3002872.s009.pdf]

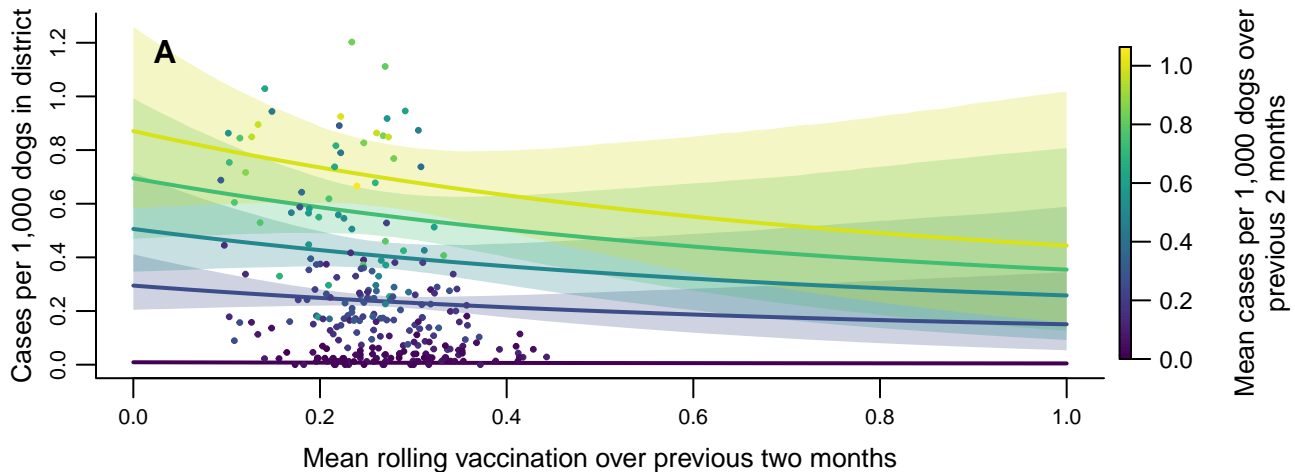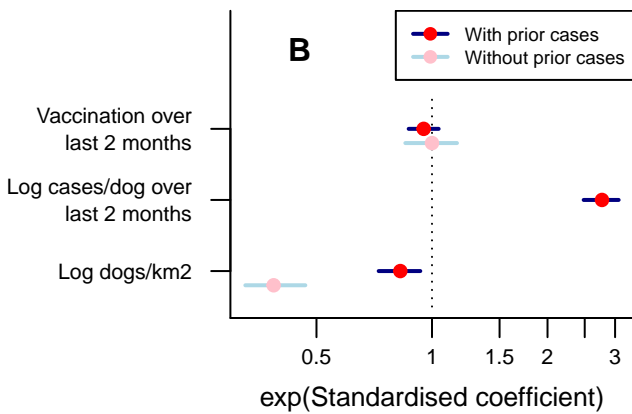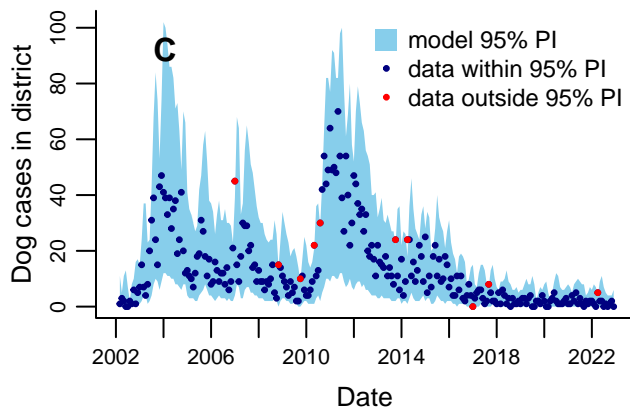

Supplement: S9 Fig — (A) Expected cases/dog (number of dog cases normalized by dog population) in the district this month for different mean rolling vaccination coverages and mean cases/dog in the prior 2 months. Shaded areas show 95% CrIs, points show the data, and predictions were obtained using the average value of dog density. (B) Exponentiated standardized values of the coefficients estimated for each explanatory variable, with 95% CrIs. Coefficients obtained for a version of the model fitted without prior cases/dog as an explanatory variable are included for comparison. See S2 Table for tabulated parameter values. (C) Comparison of observed monthly dog cases (points) with the 95% prediction interval from the fitted model. Data points in red fall outside the 95% prediction interval (PI). The data underlying this figure can be found at https://doi.org/10.5281/zenodo.15012106. (PDF) [file pbio.3002872.s010.pdf]

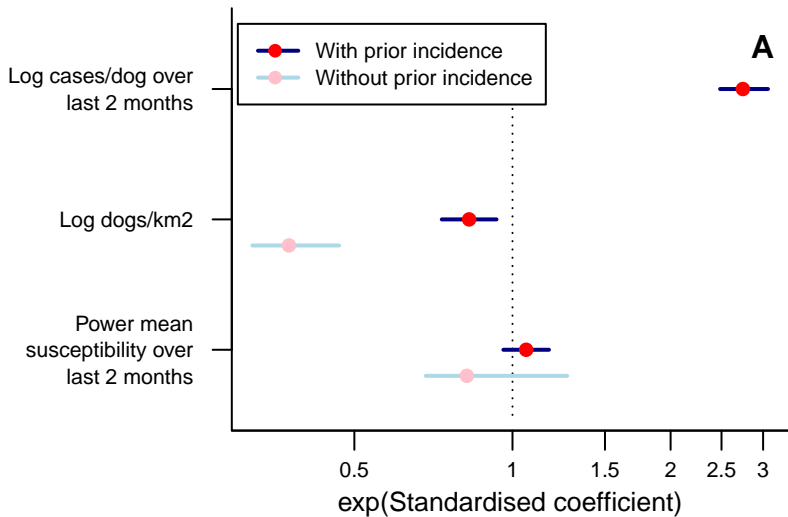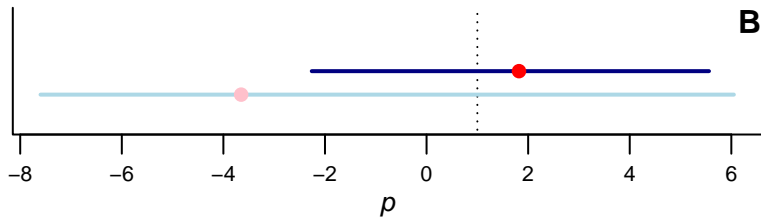

Supplement: S10 Fig — (A) Exponentiated standardized estimated coefficients for each explanatory variable, with 95% CrIs. (B) Estimated power p used to calculate power mean susceptibility. In A–B, estimates from models with and without effects of prior incidence are shown. See S2 Table for tabulated parameter values. The data underlying this figure can be found at https://doi.org/10.5281/zenodo.15012106. (PDF) [file pbio.3002872.s011.pdf]

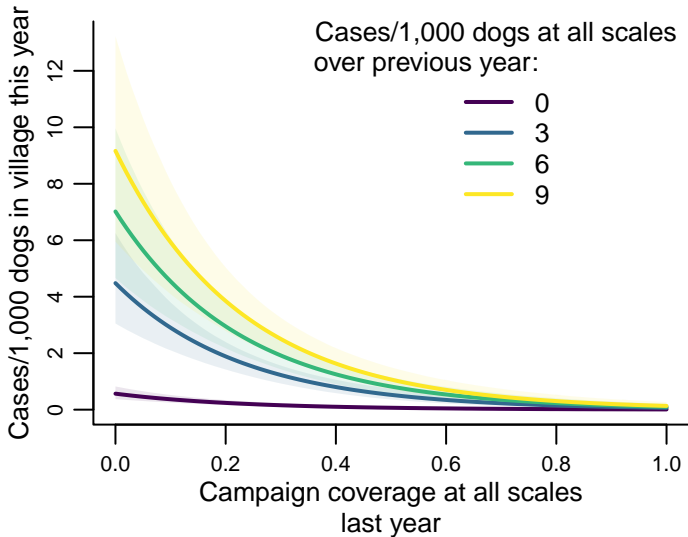

Supplement: S11 Fig — Lines show the expected cases per 1,000 dogs (number of dog cases normalized by dog population) in a village this year for different campaign vaccination coverages and cases per 1,000 dogs in the previous year. Prior incidence values were chosen to represent the range observed at district level. Shaded areas show 95% CrIs, and predictions were obtained using average values of unspecified explanatory variables. See S3 Table for tabulated parameter values. The data underlying this figure can be found at https://doi.org/10.5281/zenodo.15012106. (PDF) [file pbio.3002872.s012.pdf]

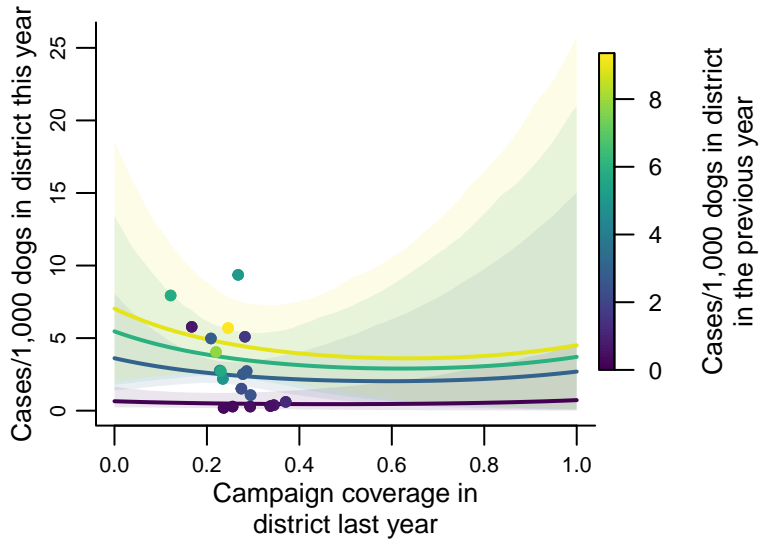

Supplement: S12 Fig — Lines show the expected cases per 1,000 dogs (number of dog cases normalized by dog population) in the district this year for different campaign vaccination coverages and cases per 1,000 dogs in the previous year. Prior incidence values were chosen to represent the range observed at district level. Shaded areas show 95% CrIs, and predictions were obtained using average values of unspecified explanatory variables. See S4 Table for tabulated parameter values. The data underlying this figure can be found at https://doi.org/10.5281/zenodo.15012106. (PDF) [file pbio.3002872.s013.pdf]

## Serial Interval Distribution

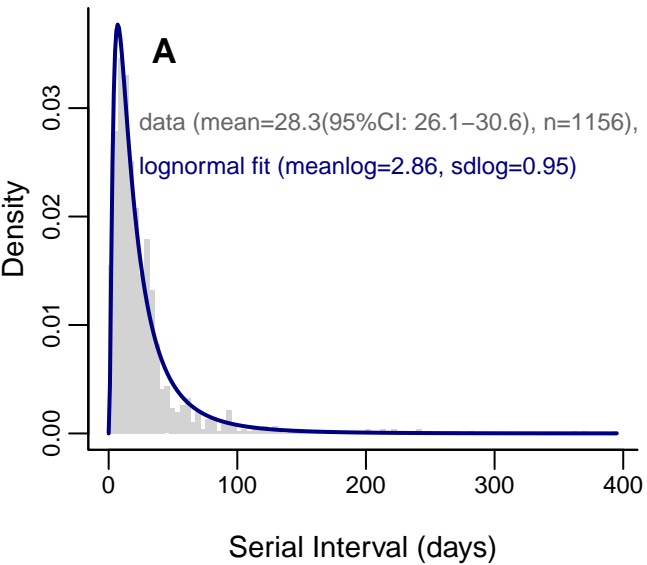

## Distance Kernel

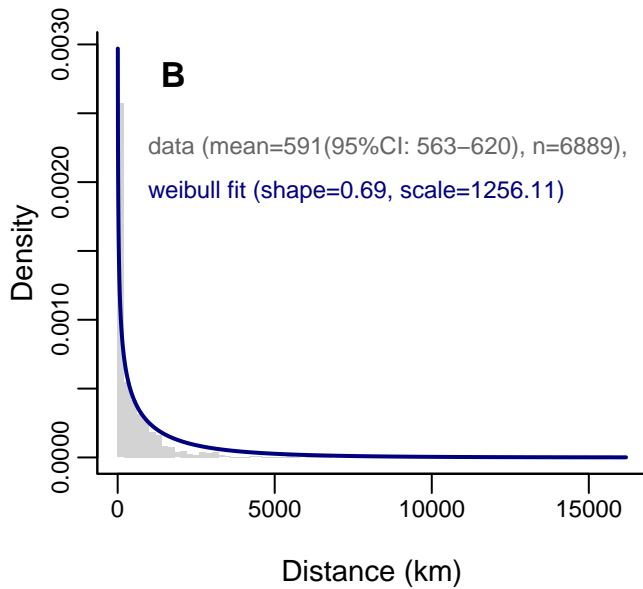

Supplement: S13 Fig — (A) Histogram of serial intervals calculated from contact tracing data (grey bars), with fitted lognormal distribution (blue line). (B) Histogram of distances between the starting location of a case and the locations of its contacts (grey bars), with fitted Weibull distribution (blue line). The data underlying this figure can be found at https://doi.org/10.5281/zenodo.15012106. (PDF) [file pbio.3002872.s014.pdf]

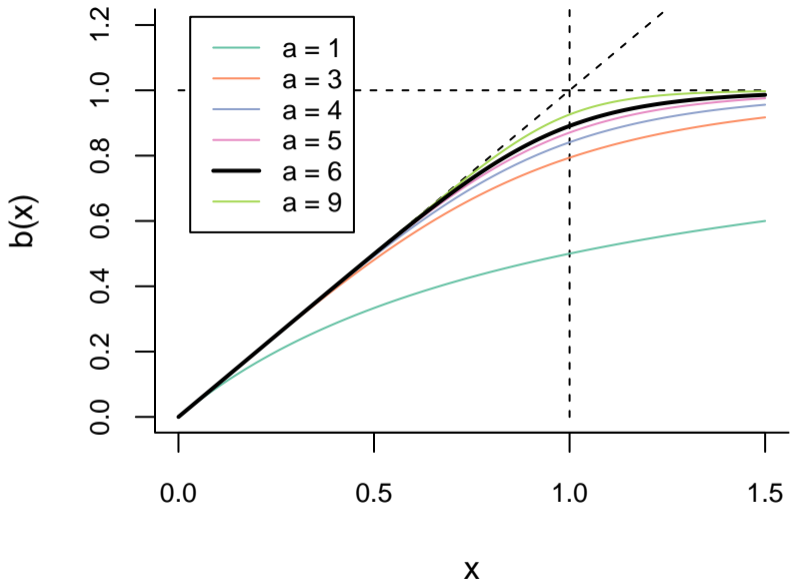

Supplement: S14 Fig — Illustration of the function b(x) = x/((1 + xa)1/a) used to bound coverage estimates below one. Throughout our analyses, we set a = 6 when applying this function, but the impact of using alternative values is shown here. The data underlying this figure can be found at https://doi.org/10.5281/zenodo.15012106. (PDF) [file pbio.3002872.s015.pdf]
