# Supplementary material for: Improved effectiveness of vaccination campaigns against rabies by reducing spatial heterogeneity in coverage
Source: PLoS Biol. 2025 May 5;23(5):e3002872. doi: 10.1371/journal.pbio.3002872 (PMC12068718; doi:10.1371/journal.pbio.3002872)
Supplement: S1 Table — Numbers of dogs that received a vaccination in each year, campaign coverage (percentage of the district dog population vaccinated in campaigns each year), and campaign completeness (percentage of villages in the district that held a campaign in each year). (DOCX) [file pbio.3002872.s016.docx]

**Table S1: Vaccination of Serengeti District by year.** Numbers of dogs that received a vaccination in each year, campaign coverage (percentage of the district dog population vaccinated in campaigns each year), and campaign completeness (percentage of villages in the district that held a campaign in each year).

|  | **2002** | **2003** | **2004** | **2005** | **2006** | **2007** | **2008** | **2009** | **2010** | **2011** | **2012** | **2013** | **2014** | **2015** | **2016** | **2017** | **2018** | **2019** | **2020** | **2021** | **2022** |
| --- | --- | --- | --- | --- | --- | --- | --- | --- | --- | --- | --- | --- | --- | --- | --- | --- | --- | --- | --- | --- | --- |
| Number of dogs that received a vaccination | 0 | 4,199 | 13,126 | 7,675 | 7,433 | 11,562 | 14,016 | 14,098 | 11,622 | 8,252 | 12,532 | 16,229 | 19,288 | 21,150 | 22,724 | 26,419 | 11,050 | 19,305 | 14,882 | 6,343 | 17,036 |
| Campaign coverage | 0 | 9 | 28 | 16 | 15 | 23 | 26 | 26 | 20 | 14 | 21 | 26 | 30 | 32 | 33 | 37 | 15 | 25 | 19 | 8 | 20 |
| Campaign completeness | 0 | 48 | 90 | 56 | 50 | 86 | 97 | 97 | 62 | 50 | 65 | 72 | 86 | 97 | 95 | 97 | 50 | 100 | 99 | 44 | 92 |
