# Supplementary material for: Improved effectiveness of vaccination campaigns against rabies by reducing spatial heterogeneity in coverage
Source: PLoS Biol. 2025 May 5;23(5):e3002872. doi: 10.1371/journal.pbio.3002872 (PMC12068718; doi:10.1371/journal.pbio.3002872)
Supplement: S2 Table — Ninety-five percent credible intervals (CrIs) in brackets. Coefficients for fixed effects where the 95% CrI does not include zero are marked *. Predictions from model 1 (and coefficients for model 2) are presented in S9 Fig). Parameters from models 3 and 4 are illustrated in S10 Fig. Vaccination, susceptibility and cases/dog variables are all averages over the prior two months. (DOCX) [file pbio.3002872.s017.docx]

**Table S2: Parameter estimates for monthly district-level GLMs for cases/dog in the district in the current month.** 95% credible intervals (CrIs) in brackets. Coefficients for fixed effects where the 95% CrI does not include zero are marked *. Predictions from model 1 (and coefficients for model 2) are presented in Fig. S9). Parameters from models 3 and 4 are illustrated in Fig. S10. Vaccination, susceptibility and cases/dog variables are all averages over the prior two months.

| **Parameter** | **1. Full model without power mean** | **2. Without power mean or prior cases/dog** | **3. Full model with power mean** | **4. Power mean without prior cases/dog** |
| --- | --- | --- | --- | --- |
| Intercept | 1.52 (0, 3.1) | 6.67 (4.03, 9.31) | 0.5 (-2.15, 2.99) | 8.01 (0.17, 11.42) |
| Rolling vaccination coverage | -0.75 (-2.06, 0.55) | -0.06 (-2.18, 2.13) |  |  |
| Susceptibility |  |  | 0.92 (-0.5, 2.46) | -1.32 (-2.98, 4.19) |
| Log cases/dog | 0.78 (0.7, 0.86)* |  | 0.78 (0.7, 0.86)* |  |
| Log dogs/km^2^ | -0.98 (-1.59, -0.38)* | -4.71 (-5.59, -3.81)* | -0.94 (-1.54, -0.35)* | -4.89 (-5.72, -3.68)* |
| size (negative binomial distribution parameter) | 4.76 (3.55, 6.32) | 1.3 (1.06, 1.57) | 4.79 (3.58, 6.31) | 1.36 (1.11, 1.65) |
| *p* (power used in calculating power means) |  |  | 2.02 (-1.74, 5.73) | -3.06 (-7.32, 6.32) |
