# Supplementary material for: Improved effectiveness of vaccination campaigns against rabies by reducing spatial heterogeneity in coverage
Source: PLoS Biol. 2025 May 5;23(5):e3002872. doi: 10.1371/journal.pbio.3002872 (PMC12068718; doi:10.1371/journal.pbio.3002872)
Supplement: S3 Table — Ninety-five percent credible intervals in brackets. Coefficients for fixed effects where the 95% CrI does not include zero are marked *. Predictions from the model 4 (including campaign coverage and cases/dog in the last year as explanatory variables) are presented in S11 Fig. (DOCX) [file pbio.3002872.s018.docx]

**Table S3: Coefficients for the annual village-level negative binomial GLMMs.** 95% credible intervals in brackets. Coefficients for fixed effects where the 95% CrI does not include zero are marked *. Predictions from the model 4 (including campaign coverage and cases/dog in the last year as explanatory variables) are presented in Fig. S11.

| **Coefficient** | **1. Model with campaign coverage last year** | **2. Model with mean campaign coverage over the last 2 years** | **3. Model with mean campaign coverage over the last 3 years** | **4. Model with campaign coverage and incidence last year** |
| --- | --- | --- | --- | --- |
| Intercept | 1.37 (-0.96, 3.98) | -0.02 (-2.35, 2.73) | -0.62 (-2.97 - 2.17) | 1.5 (-0.03, 3.13) |
| Campaign coverage last year in the focal village | -1.12 (-1.8, -0.43)* |  |  | -1.07 (-1.73, -0.38)* |
| Campaign coverage last year in bordering villages | -0.44 (-1.54, 0.65) |  |  | -0.5 (-1.56, 0.56) |
| Campaign coverage last year in non-bordering villages | -0.81 (-2.35, 0.66) |  |  | -2.73 (-4.09, -1.37)* |
| Mean campaign coverage over the last 2 years in the focal village |  | -1.32 (-2.25, -0.34)* |  |  |
| Mean campaign coverage over the last 2 years in bordering villages |  | -1.38 (-2.92, 0.15) |  |  |
| Mean campaign coverage over the last 2 years in non-bordering villages |  | -1 (-3.08, 1.08) |  |  |
| Mean campaign coverage over  the last 3 years in the focal village |  |  | -1.48 (-2.63, -0.29)* |  |
| Mean campaign coverage over  the last 3 years in bordering villages |  |  | -1.65 (-3.51, 0.2) |  |
| Mean campaign coverage over  the last 3 years in non-bordering villages |  |  | -0.42 (-3.28, 2.45) |  |
| Log cases/dog last year in the focal village |  |  |  | 0.08 (0.02, 0.14)* |
| Log cases/dog last year in bordering villages |  |  |  | 0.16 (0.07, 0.26)* |
| Log cases/dog last year in non-bordering villages |  |  |  | 0.43 (0.29, 0.56)* |
| Log dogs/km^2^ | -2.9 (-3.67, -2.2)* | -2.44 (-3.29, -1.68)* | -2.28 (-3.22, -1.52)* | -1.37 (-1.9, -0.92)* |
| Human:dog ratio | 0.53 (0.22, 0.85)* | 0.56 (0.28, 0.87)* | 0.58 (0.28, 0.87)* | 0.38 (0.18, 0.58)* |
| Standard deviation of village random effect | 1.69 (1.28, 2.16) | 1.5 (1.11, 1.97) | 1.44 (1.05, 1.94) | 1 (0.76, 1.3) |
| size (negative binomial distribution parameter) | 0.43 (0.38, 0.49) | 0.41 (0.36, 0.47) | 0.38 (0.33, 0.44) | 0.49 (0.42, 0.55) |
