# Supplementary material for: Improved effectiveness of vaccination campaigns against rabies by reducing spatial heterogeneity in coverage
Source: PLoS Biol. 2025 May 5;23(5):e3002872. doi: 10.1371/journal.pbio.3002872 (PMC12068718; doi:10.1371/journal.pbio.3002872)
Supplement: S4 Table — Ninety-five percent credible intervals in brackets. Coefficients for fixed effects where the 95% CrI does not include zero are marked *. Predictions from model 4 (including campaign coverage and cases/dog in the last year as explanatory variables) are presented in S12 Fig. (DOCX) [file pbio.3002872.s019.docx]

T**able S4: Coefficients for the annual district-level negative binomial GLMs.** 95% credible intervals in brackets. Coefficients for fixed effects where the 95% CrI does not include zero are marked *. Predictions from model 4 (including campaign coverage and cases/dog in the last year as explanatory variables) are presented in Fig. S12.

| **Coefficient** | **1. Model with campaign coverage last year** | **2. Model with mean campaign coverage over the last 2 years** | **3. Model with mean campaign coverage over the last 3 years** | **4. Model with campaign coverage and incidence last year** |
| --- | --- | --- | --- | --- |
| Intercept | 12.29 (4.84, 19.74) | 13.8 (5.6, 22.2) | 14.64 (5.61, 23.25) | 9.51 (4, 15.09) |
| Campaign coverage last year | 0.55 (-4.1, 4.93) |  |  | -1.08 (-4.66, 2.42) |
| Mean campaign coverage over the last 2 years |  | 0.6 (-5.75, 6.87) |  |  |
| Mean campaign coverage over  the last 3 years |  |  | 1.92 (-6.96, 10.67) |  |
| Log cases/dog last year |  |  |  | 0.55 (0.21, 0.89)* |
| Log dogs/km^2^ | -5.7 (-8.04, -3.29)* | -6.15 (-8.79, -3.45)* | -6.5 (-9.16, -3.6)* | -3.72 (-5.84, -1.64)* |
| size (negative binomial distribution parameter) | 2.08 (1.03, 3.6) | 2.08 (0.99, 3.59) | 2.03 (0.94, 3.56) | 3.25 (1.5, 5.81) |
