# Supplementary material for: Improved effectiveness of vaccination campaigns against rabies by reducing spatial heterogeneity in coverage
Source: PLoS Biol. 2025 May 5;23(5):e3002872. doi: 10.1371/journal.pbio.3002872 (PMC12068718; doi:10.1371/journal.pbio.3002872)
Supplement: S5 Table — For each epidemiological variable calculated from the contact tracing data with sample size n, we fitted gamma, lognormal and Weibull distributions. Estimates of the parameters for each distribution are provided. The best fitting model for each epidemiological variable based on AIC is highlighted in bold. The 95th, 97.5th and 99th percentiles of each distribution are given. (DOCX) [file pbio.3002872.s020.docx]

**Table S5: Fitted distributions for incubation period (units=days), serial interval (units=days) and distance kernel (units=metres).** For each epidemiological variable calculated from the contact tracing data with sample size n, we fitted gamma, lognormal and Weibull distributions. Estimates of the parameters for each distribution are provided. The best fitting model for each epidemiological variable based on AIC is highlighted in bold. The 95th, 97.5th and 99th percentiles of each distribution are given.

|  | **n** | **Distribution** | **Parameter 1: Name** | **Parameter 1: Value (95%CI)** | **Parameter 2: Name** | **Parameter 2: Value (95%CI)** | **AIC** | **95th percentile** | **97.5th percentile** | **99th percentile** |
| --- | --- | --- | --- | --- | --- | --- | --- | --- | --- | --- |
| **Incubation Period** | 1,212 | Gamma | shape | 1.18 (1.1, 1.26) | rate | 0.04 (0.04, 0.05) | 10421 | 77.2 | 93.9 | 115.9 |
|  |  | **Lognormal** | **meanlog** | **2.82 (2.77, 2.87)** | **sdlog** | **0.95 (0.91, 0.99)** | **10159** | **80.1** | **108.0** | **152.9** |
|  |  | Weibull | shape | 1 (0.96, 1.05) | scale | 27.27 (25.78, 28.89) | 10440 | 81.6 | 100.5 | 125.4 |
| **Serial Interval** | 1,156 | Gamma | shape | 1.18 (1.1, 1.27) | rate | 0.04 (0.04, 0.05) | 10022 | 79.9 | 97.2 | 119.9 |
|  |  | **Lognormal** | **meanlog** | **2.86 (2.81, 2.92)** | **sdlog** | **0.95 (0.91, 0.99)** | **9781** | **83.4** | **112.5** | **159.3** |
|  |  | Weibull | shape | 1 (0.96, 1.05) | scale | 28.29 (26.73, 30.09) | 10041 | 84.5 | 104.0 | 129.8 |
| **Distance kernel** | 6,897 | Gamma | shape | 0.58 (0.55, 0.6) | rate | 0.00038 (0.00035, 0.00041) | 45177 | 5596.7 | 7216.4 | 9407.9 |
|  |  | Lognormal | meanlog | 6.45 (6.4, 6.49) | sdlog | 1.74 (1.69, 1.8) | 45321 | 11129.8 | 19284.6 | 36540.9 |
|  |  | **Weibull** | **shape** | **0.69 (0.67, 0.71)** | **scale** | **1253.12 (1192.2, 1315.93)** | **45143** | **6142.8** | **8304.8** | **11453.2** |
